# Prediction of tumor location in prostate cancer tissue using a machine learning system on gene expression data

**DOI:** 10.1186/s12859-020-3345-9

**Published:** 2020-03-11

**Authors:** Osama Hamzeh, Abedalrhman Alkhateeb, Julia Zheng, Srinath Kandalam, Luis Rueda

**Affiliations:** 1 School of Computer Science, University of Windsor, 401 Sunset Ave, Windsor, N9B 3P4 ON Canada; 2 Department of Biomedical Sciences, University of Windsor, 401 Sunset Ave, Windsor, N9B 3P4 ON Canada

**Keywords:** Machine learning, Classification, Biomarkers, Prostate cancer laterality

## Abstract

**Background:**

Finding the tumor location in the prostate is an essential pathological step for prostate cancer diagnosis and treatment. The location of the tumor – the laterality – can be unilateral (the tumor is affecting one side of the prostate), or bilateral on both sides. Nevertheless, the tumor can be overestimated or underestimated by standard screening methods. In this work, a combination of efficient machine learning methods for feature selection and classification are proposed to analyze gene activity and select them as relevant biomarkers for different laterality samples.

**Results:**

A data set that consists of 450 samples was used in this study. The samples were divided into three laterality classes (left, right, bilateral). The aim of this work is to understand the genomic activity in each class and find relevant genes as indicators for each class with nearly 99% accuracy. The system identified groups of differentially expressed genes (RTN1, HLA-DMB, MRI1) that are able to differentiate samples among the three classes.

**Conclusion:**

The proposed method was able to detect sets of genes that can identify different laterality classes. The resulting genes are found to be strongly correlated with disease progression. HLA-DMB and EIF4G2, which are detected in the set of genes can detect the left laterality, were reported earlier to be in the same pathway called Allograft rejection SuperPath.

## Background

Cancer is among the leading causes of death worldwide. In 2013, there were 8.2 million deaths, and 14.9 million cases of cancer incidence [1]. As with all cancer diseases, investigating prostate cancer at the molecular level reveals transcriptional and regulatory mechanisms of the tumour biology. Traditionally, prostate cancer studies centered primarily on finding biomarkers for differentiation between benign and cancerous tumors. Recently, studies have considered some other aspects of the tumours including progression, metastasis, location, and recurrence, among others.

Traditional methods for detecting prostate cancer such as prostate specific antigen (PSA) blood test, transrectal ultrasound image (TRUS) guided biopsy, and digital rectal exam (DRE) do not measure up to the medical standards. PSA blood test statistical results shows a specificity of 61% and a low sensitivity of 34.9%, while TRUS-guided biopsy and DRE are invasive [2].

In addition, multiparametric magnetic resonance imaging (MRI) of the prostate is a functional form of imaging used to augment standard T1- and T2-weighted imaging. Multiparametric MRI may miss up to 12% of cancer cases [3]. In addition to the need for reducing the number of biopsies come most of the time with pain, fever, bleeding, infection, transient urinary difficulties, or other complications that require hospitalization [4]. Finding gene biomarkers of prostate cancer location and analyzing their proteomics can help clinically understand the development of the disease and improve treatment efficiency.

Machine learning approaches, on the other hand, have been successfully applied on prostate cancer data to identify gene biomarkers of the disease [5]. Using next generation sequencing and the power of machine learning, alkhateeb et al. devised a support vector machine (SVM) classifier to identify biomarker genes associated with prostate cancer progression. The biomarkers were able to discriminate consecutive prostate cancer stages with high performance [5]. Earlier, Hamzeh et al. proposed a method for finding groups of transcripts that are differentially expressed among the different Gleason stages [6]. The identified transcripts can be used to predict the actual Gleason score for new samples, and these transcripts belong to genes that are well known to play important roles in prostate and other types of cancer. Yu et al. demonstrated that their method is efficient for predicting prostate cancer aggressiveness based on gene expression patterns [7].

Similarly, machine learning approaches have been used for cancer localization prediction [8, 9]. Artan et al. proposed a prediction model based on a cost-sensitive SVM. The model is used to analyze a large data set of multispectral magnatic resonance imaging (MRI). This method improves the cost-sensitive SVM using a segmentation method by combining conditional random fields (CRF) with a cost-sensitive framework. Incorporating spatial information leads to better localization accuracy [8]. As stated earlier, prediction by imaging is still inaccurate, not specific and hence needs more improvement. In an attempt to find different gene expression levels between two lists, the first contains the expression levels of colon tumor cells, while the latter for rectal tumor cells, Sanz-Pamplona et al. applied agglomerative hierarchical clustering to display the classification ability between both lists. Both lists have very similar gene expression levels except for several HOX genes which are found to be associated with tumor location [9].

In this work, we are extending our previous method for classifying different laterality prostate samples which are left unary, right unary, or bilateral [10]. The results of this multi-class model are set of genes that can determine a specific class from the others. The literature shows that these genes are related to prostate cancer, which may lead to be a potential biomarkers for prostate cancer laterality.

## Materials and methods

**Fig. 1:**
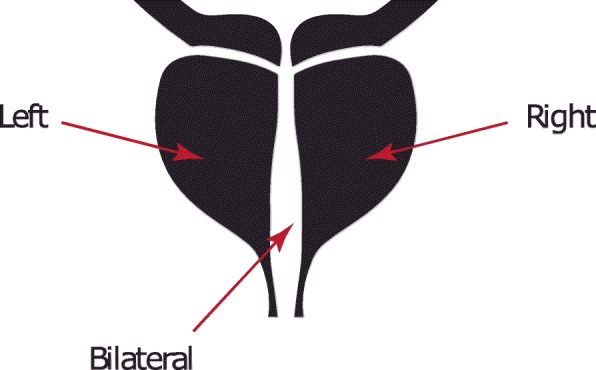
Possible locations of the tumor in prostate cancer

RNA-sequencing data from The Cancer Genome Atlas (TCGA) Prostate Adenocarcinoma (PRAD) was used. This data set consists of 450 samples for different patients with different cancer locations. There are three primary locations that the tumor might be located within the prostate: left, right and bilateral. Figure 1 shows the actual possible locations, while Table 1 describes the number of samples in each location.

**Table 1:** Number of samples in each prostate cancer tumor location

Left	Bilateral	Right
18	431	38

Gene expression data was downloaded through the cBioPortal for cancer genomics database [11]. Each sample contains expression levels for each of the 60,488 genes; the gene expressions are given in terms of Transcripts Per Kilobase Million (TPM) values. The aim of this study is to identify genes which are associated with specific tumor locations, and hence we need to use the genes as features and the actual locations as classes to build a model to predict locations for future samples. Since most of the samples are bilateral, we deal with a class imbalance problem. We used the re-sampling method proposed in [12] as measure to lower the effect of this imbalance. The gene expressions of each node in the classification models are included in Additional file 1 that contains Bilateral-vs-Rest gene’s expressions, Additional file 2 that contains Left-vs-Rest gene’s expressions and Additional file 3 that contains Right-vs-Rest gene’s expressions.

### Re-sampling

By observing Table 1, we clearly notice that there is a class imbalance problem, where the number of samples in the right class (38) is almost twice as large as that of the left class (18). while the number of samples of the bilateral class (431) is more than twenty times larger than the left class and more than ten times larger than the right class.

To solve this problem, multiple re-sampling methods were deployed and tested to identify a method that would yield the best solution for our data set. Oversampling provides a fast solution for classes left and right. This method duplicates samples from the minority classes and adds them until yielding a similar number of samples for each class. Applying oversampling directly did resolve the class imbalance problem and provided high accuracy for classifiers, although after taking a closer look at the samples used in these classifiers, we noticed that there was a major over-fitting. Based on the literature [13, 14], we selected the combination of oversampling Synthetic Minority Oversampling Technique (SMOTE) [15] and Neighborhood Cleaning Rule (NCL) [16] for under-sampling the majority class. Junsomboon et al. reported that the combination (NCL + SMOTE) outperformed another set of methods for handling the imbalance data sets. They have applied this combination on different health related data sets [13]. NCL uses the Wilson’s Edited Nearest Neighbor Rule (ENN) to remove majority class outliers [17]. Batista et al. reported a high performance for SMOTE + ENN in handling imbalance data set [14].

**Fig. 2:**
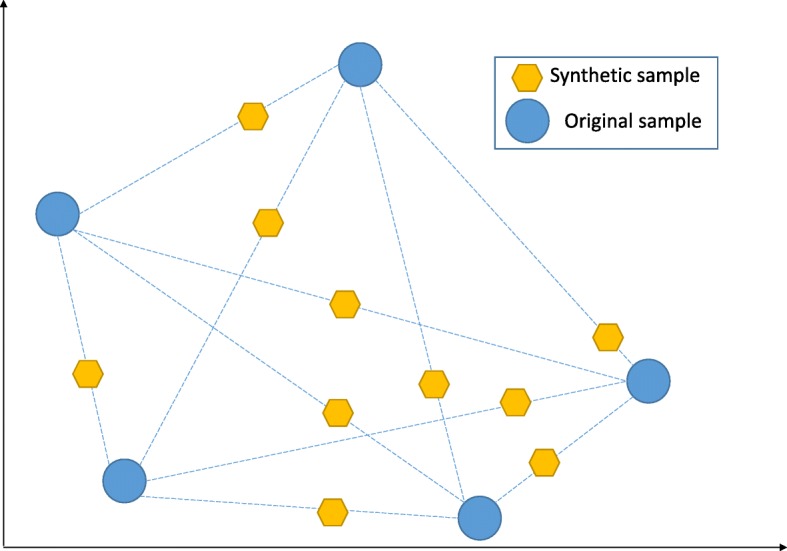
Synthetic Minority Oversampling Technique (SMOTE) works by adding new synthetic sample randomly along the line that connects each of the two original samples

NCL works by removing any sample whose class is different from the class of at least two of its three nearest neighbors. SMOTE introduces a new way of creating new samples, by utilizing the feature vector connecting each sample and introducing a new synthetic sample along the line that connects the two underlying samples. The exact location of the new sample on the line itself is calculated by measuring the distance between the two samples and multiplying that value by a random number between 0 and 1. Figure 2 shows the behavior of SMOTE.

Applying these two methods allowed us to use three classes that are balanced. Table 2 shows the number of samples after applying the SMOTE + ENN re-sampling methods.

**Table 2:** Number of samples in each prostate cancer tumor location after applying the SMOTE + ENN resampling methods

240	240	38	38	240	240
Left vs Bilateral		Left vs Right		Right vs Bilateral	

### Feature selection

Dealing with a huge number of features lead us to the problem of curse of dimensionality. As such, we use machine learning techniques to lower the number of features used for classification. We applied the information gain (IG) feature selection method [18] to rank all the genes with a score that relates to the highest information gain against the different classes. We then chose the attributes with the highest scores, discarding those with lower scores. In this paper, the IG attribute evaluator [18] is used to evaluate each attribute. IG of feature *X* with respect to class *Y* is calculated as follows:

1 $$ IG(Y,X) = H(Y) - H(Y\vert{}X)  $$

where,

2 $$ H(Y) = - \sum_{y\in{}Y}p(y){log}_{2}(p(y)).  $$

and

3 $$ H(Y\vert{}X) = - \sum_{x\in{}X}p(x)\sum_{y\in{}Y}p(y\vert{}x){log}_{2}(p\left(y\vert{}x\right)).  $$

Here, *H(Y)* is the entropy of class *Y* and *H(Y |X)* is the conditional entropy of *Y* given *X*.

The next step is to choose the best set of attributes (genes) that provide good classification among the different classes.

A wrapper that binds feature selection and classification methods is used. The feature selection method is the minimum redundancy maximum relevance (mRMR), which takes features that contain minimum redundancy while at the same time have high correlation to the classification variable [19]. The equation for minimizing redundancy (*W*_*i*_) and maximizing the relevancy (*V*_*i*_) is the following:

4 $$ min\, W(S),\,W=\frac{1}{\vert{}S{\vert{}}^{2}}\ \sum_{i,j\in{}S}I(i,j),  $$

and

5 $$ max\,V(S,h),\,V=\frac{1}{\vert{}S\vert{}}\ \sum_{i\in{}S}I(h,i),  $$

Where *S* is the set of features, *I(i,j)* is mutual information between features *(i,j)*, *h* is the class.

The operator *ϕ*(W,V) is defined to combine W and V and consider the following simplest form to optimize W and V at the same time:

6 $$ max\,\phi(D,R),\phi=W-V,  $$

### Classification

We deal with a multi-class classification problem which is solved by using the one-versus-all approach. We have three different classes which are the three different locations. To apply the one-versus-all approach, we need to create three separate copies from the actual data set. For each data set, we set one of the classes to positive, and the rest of the classes are combined together to form the negative class. We used accuracy, sensitivity and specificity to choose the best classification method.

Multiple classification methods were applied on the data to identify which methods separate the locations better. Accordingly, the probabilistic classifier Naive Bayes that applies Bayes’ theorem with the assumption of independence between the features [20] was tested. SVM was also used to build a classification model based on the features selected in the previous step [21]. The other classifier that was tested is random forest [22], which attempts to build multiple decision tree models with different samples and different initial variables.

The Weka open source tool were used to run different classification algorithms on the minimized number of features to identify which genes are differentially expressed in the different locations [23].

## Results and discussion

**Fig. 3:**
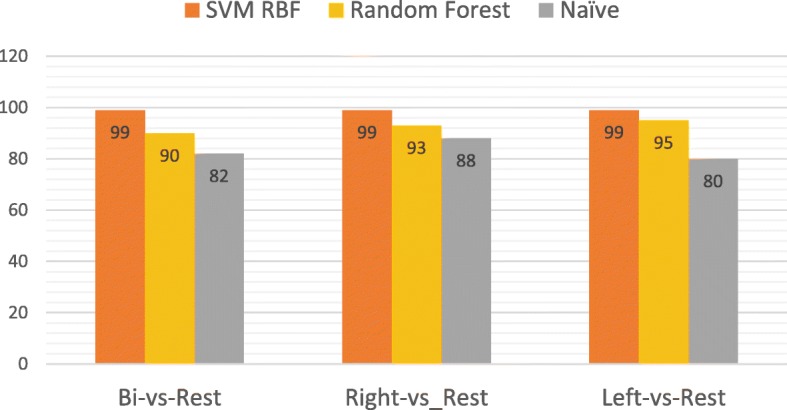
Different classifiers accuracy for the different locations

The different classifiers produced varied results as observed in Table 3 and Fig. 3. The classifiers were chosen based on the accuracy and the precision, as leading high accuracy with low precision is not a good criterion at all. The accuracy measures the number of correctly classified samples divided by the number of all samples, while the precision is the true positive rate which measures the number of true positive calls divided by all positive calls. Table 3 shows the actual accuracy and precision for each classifier. The highest accuracy and precision for the different classifiers came from the SVM Radial basis function kernel (SVM-RBF) classifier. Grid search optimization was applied to fine tune the RBF classifier, it was able to separate the different locations by an accuracy of 99%. Random forest managed to result in high accuracy too, while the naive Bayes classifier results were not satisfactory.

**Table 3:** Accuracy and precision for classifying each class versus the rest

Classifier	Accuracy	Precision	Accuracy	Precision	Accuracy	Precision
SVM RBF	99	97	99	97	99	97
Naive Bayes	88	78	82	78	80	78
Random Forest	93	85	90	85	95	85
	Left vs rest		Bilateral vs rest		Right vs rest	

Table 4 show the actual genes that were identified by SVM-RBF. These genes can be used to predict the location of the prostate cancer tumor very accurately from gene expression data.

**Table 4:** Genes that can predict tumors in each location class of the prostate tumor

Ensemble	Gene	Ensemble	Gene	Ensemble	Gene
ENSG00000135108.13	FBXO21	ENSG00000120697.7	ALG5	ENSG00000242574.7	HLA-DMB
ENSG00000139970.15	RTN1	ENSG00000279453.1	Z99129	ENSG00000124193.13	SRSF6
ENSG00000128609.13	NDUFA5	ENSG00000019549.7	SNAI2	ENSG00000110321.14	EIF4G2
ENSG00000172336.4	POP7	ENSG00000037757.12	MRI1		
		ENSG00000178913.7	TAF7		
Left vs rest		Bilateral vs rest		Right vs rest	

Throughout our model 10-fold cross-validation was used. The proposed method identified 12 genes that are differentially expressed among the three different possible locations.

It is important to highlight that most of the genes identified in this work have been previously characterized and described to play some role in prostate cancer as well as other types of cancer. SNAI2 is a gene shown [24] to be silenced in prostate cancer and regulates neuroendocrine differentiation, metastasis-suppressor, and pluripotency gene expression.

Likewise, the results shown in [25, 26] indicate that increased TAF1/7 expression is associated with progression of human prostate cancers to the lethal castration-resistant state. In a similar way, the results reported in [27] found that tumor cell expression of HLA-DMB is associated with increased numbers of tumor-infiltrating CD8 T lymphocytes and both are associated with improved survival in advanced serous ovarian cancer.

**Fig. 4:**
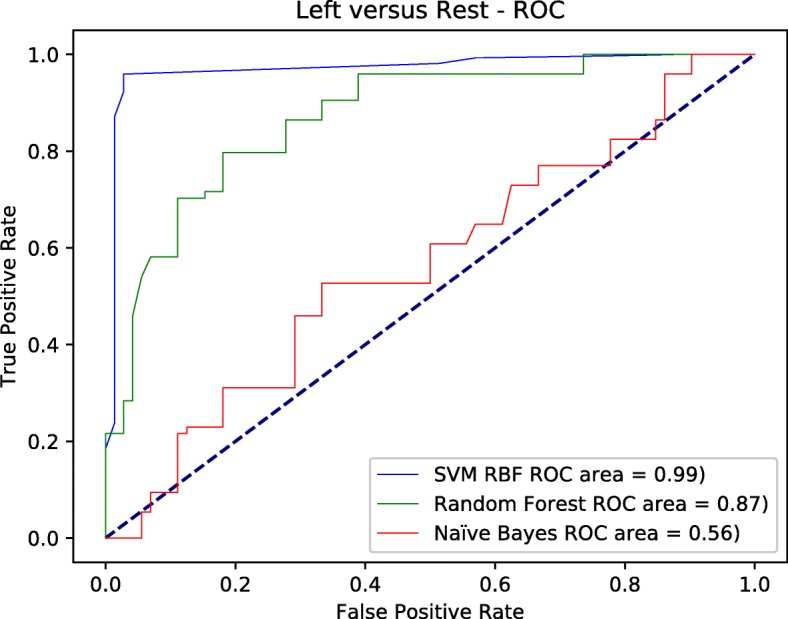
The ROC curve for left versus the rest using different classifiers

**Fig. 5:**
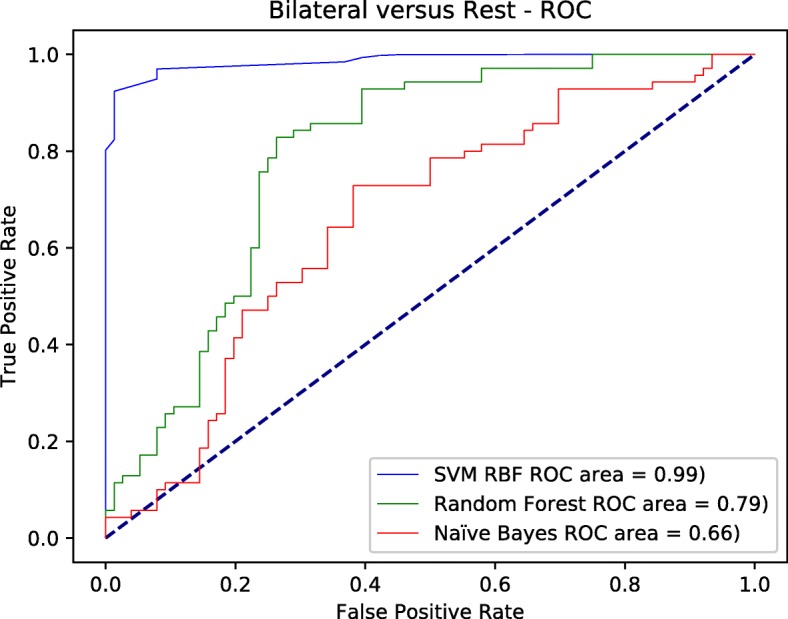
The ROC curve for belateral versus the rest using different classifiers

**Fig. 6:**
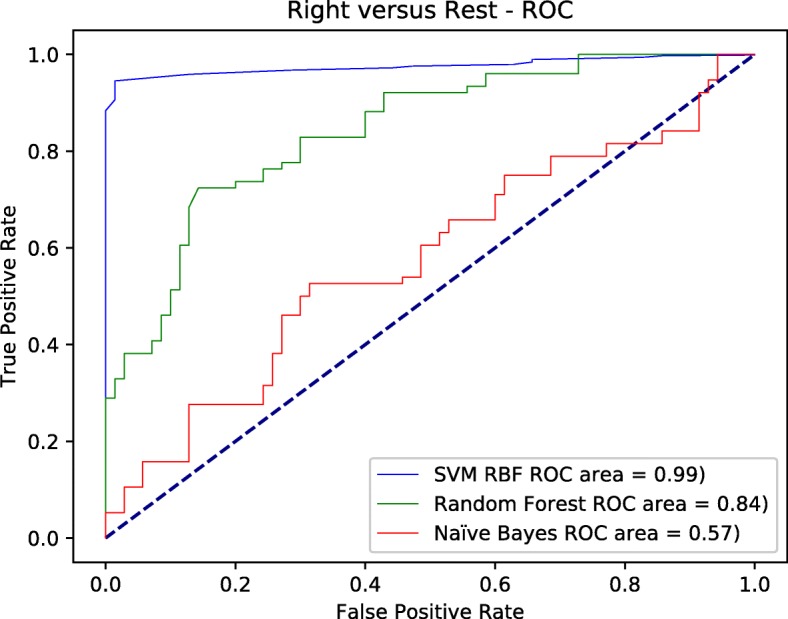
The ROC curve for right versus the rest using different classifiers

Figures 4, 5, and 6 depict the ROC curves for all the classes versus the rest at each node. The area under the curve AUC for SVM-RBF tends to be further towards the north west with 0.99 value in the three figures, which means the best overall performance across all classes versus the rest. All other classifiers were inconsistent in the three figures. However, random forest performed very well in later false positive rates for both left and right classes with overall performance 0.87, 0.84 in order for both classes. it slightly outperformed the SVM-RBF in one point at both classes. but as we stated earlier, it was inconsistent through out different running parameters for false positive rates.

### Biological insight

We have conducted a thoroughly literature review on the most up to date classification, as well as in the relevant databases and gathered valuable information about the most relevant genes that we have found in our study. A summary for each gene is given below and opens the avenue for future studies as well as additional lab experiments that can corroborate our studies and lead to novel ways of diagnosis, treatment and prognosis of the disease.

FBXO21 (F-box protein 21) is part of the multi-protein complex, SCF E3-ligase, which functions in phosphorylation-dependent ubiquitination. FBXO21 may affect prostate cancer through different mechanisms, and here we hypothesize two possibilities. Firstly, ABCB1 is a known tumour drug resistance biomarker because it is a multi-drug efflux pump linked with the development of metastases [28]. FBXO21 tags ABCB1 for proteasomal degradation, whereas inhibition of FBXO21 leads to higher expression level of ABCB1. Secondly, FBXO21 recognizes EID1 in cycling and G0 stage cells and targets it for degradation. EID1 interacts with retinoblastoma tumour suppressor (pRB), melanoma-associated antigen (MAGE), and E1A binding protein p300 (EP300) as well as being involved in the coupling cell cycle exit to cellular differentiation. All available evidence suggests that FBXO21 may be down-regulated in prostate cancer, although further research is desirable [29].

RTN1 (reticulon 1) is associated with the endoplasmic reticulum (ER) and is involved in neuroendocrine secretions and membrane trafficking. RTN1 has been known exert a cancer-specific proapoptotic function. Specifically, RTN1-C regulates the two mutually exclusive ER stress-induced apoptosis and DNA damage-induced cell death. Over-expression of RTN1-C results in ER stress-induced cell death mediated by aberrantly increased cytosolic *Ca*^2+^ due to depletion of ER calcium stores [30]. A recent publication on prostate cancer shows that silencing RTN1 by siRNA enabled androgen-independent proliferation of androgen-dependent prostate cancer tumours. The knockdown of RTN1 increases the nuclear concentration of HDAC8, a multi-functional histone deacetylase that regulates activity of transcription factors such as nuclear hormone receptors [31]. In particular, it is known that ceramide inhibits androgen receptor activity and inhibits androgen-independent growth by activation of protein phosphatase 2A (PP2A) [32]. However, HDAC8-induced depletion of SPTSSA in the ER compromises the ER-localized ceramide biosynthesis pathway, leading to down-regulation of ceramide, partial inhibition of PP2A and androgen receptor activation in androgen-deprived conditions [31]. Consequently, RTN1 may be a proto-oncogene associated with aggressive, malignant and androgen-independent prostate cancer.

NDUFA5 (NADH:ubiquinone oxidoreductase subunit A5) is localized to the inner mitochondrial membrane and functions in the NADH two-electron reduction of ubiquinone [33]. Complex I, also known as NADH-ubiquinone oxidoreductase, is the first complex of the mitochondrial oxidative phosphorylation (OXPHOS) system. The energy released is coupled with generation of the electrochemical gradient necessary for ATP synthesis [34]. As expected, NDUFA5 activity is lower in hypoxic cells [35]. The Warburg effect states that tumour cells demonstrate drastically increased glycolysis activity compared to oxidative phosphorylation due to target genes up-regulated by hypoxia-inducible factor (HIF) [36]. On the other hand, NDUFA5 is up-regulated in HPV+ cervical cancer and its over-expression may play a role in carcinogenesis through acquiring growth advantage and resistance against an apoptotic signal [33]. In a recent publication, NDUFA5 also gained copy numbers in both low-grade and high-grade gliomas. Therefore, NDUFA5 may also be up-regulated in prostate cancer, although further research is necessary to confirm this hypothesis [37].

POP7 (POP7 homolog, ribonuclease P/MRP subunit) is discovered in S. cerevisiae. POP7 heterodimerizes to POP6 and binds to the P3 domain of catalytic ribonucleoproteins RNase MRP (mitochondrial RNA processing) and Rpr1 RNA [38]. RNase MRP is critically important to the viability of eukaryotic cells because it is localized in the nucleolus and is involved in processing mitochondrial RNAs and regulating mitochondrial DNA replication [39]. POP1/POP6/POP7 complex is required for telomere elongation protein (Est1) to associate with the RNP, which is critical during the process of mitosis for the cell lifespan before its senescence [40]. Despite the critical importance of POP7, no known human diseases are associated with this gene currently. Further research will be important to explore the biological significance of POP7.

HLA-DMB (major histocompatibility complex class II, DM beta) is a sub-unit of the HLA class II heterodimer found embedded in intracellular vesicles. In antigen-presenting cells (APC), HLA-DMB is critical in the antigen-presentation machinery by releasing class II-associated invariant chain peptide (CLIP) from MHC class II molecules so that the peptide binding site is free to interact with antigenic peptides [41]. A recent publication on prostate cancer research found that HLA-DMB is co-expressed with ERG and silencing ERG led to significant under-expression of HLA-DMB. Thus, HLA-DMB is an up-regulated tumour-associated gene in prostate cancer [42].

SRSF6 (Serine and Arginine rich Splicing Factor 6) modulates a splicing factor protein called SFRS12 to determine alternative splicing of mRNA. In a recent publication on colorectal cancer, SRSF6 targeted ZO-1 (tight junction protein 1) exon23 for alternative splicing, consequentially disrupting ZO-1 from regulating tight junctions between adjacent cells [43]. Furthermore, SRSF6 is the direct target of LINC01133, a key SRSF6 modulates a splicing factor protein called SFRS12 to determine alternative splicing of mRNA. In a 2017 paper on colorectal cancer, SRSF6 targeted ZO-1 (tight junction protein 1) exon23 for alternative splicing, consequentially disrupting ZO-1 from regulating tight junctions between adjacent cells. In addition, SRSF6 is the direct target of LINC01133, a key downstream protein of TGF- *β* signaling pathway which is critical for cell growth and differentiation [44]. Silencing SRSF6 in colorectal cancer tissues inhibited epithelial-mesenchymal transition, tissue invasion, and metastasis. A study on wound healing found that over-expression of SRSF6 induces skin hyperplasia due to SRSF6 up-regulating Tenascin C and suppressing the normal epithelial differentiation mechanism. Therefore, SRSF6 may be up-regulated in prostate cancer [43].

EIF4G2 gene, Eukaryotic Translation Initiation Factor 4 Gamma 2 is a cap - binding protein complex which has three sub units – eiF4A, eiF4E eiF4G. The gene is known to up-regulate p21, a cyclin dependant kinase inhibitor and interleukin 6 [45]. Higher expression levels of p21 oncogene protein are found with increasing prostate cancer tumor grade [46]. Interleukin 6 is involved in the progression of prostate cancer [47], and is used as a clinicopathological feature by detecting the levels in serum [48]. With the up-regulated expression levels of EIF4G2 gene in prostate cancer, it can be used as a potential marker for studying the progression of the disease.

Interestingly, EIF4G2 and HLA-DMB which are part of the gene set that can identify right side from the rest, they are both part of Allograft rejection SuperPath pathway [49].

The discovery of fusion protein transcripts in the recent times have helped studying prostate cancer development with much detail. ALG5, Dolichyl-Phosphate Beta-Glucosyltransferase and PIGU, Phosphatidylinositol Glycan Anchor Biosynthesis Class forms a chimeric-fusion protein transcript in which glucosyltransferase, the head from ALG5 is retained but GPI transamidase, the tail has been eliminated in PIGU resulting in the loss of functionality of both the genes [50]. The uncommon joining of the genes would result in serious complications in the overall environment of the cell causing further progression of the cancer. The transcription of the fused ALG5-PIGU is androgen independent [51]. Fusion protein transcripts will serve as an important biomarker both in detection and treatment of Prostate Cancer.

SNAI2, Snail Family Transcriptional Repressor 2 encodes zinc-finger protein of the Snail family transcription factors, is involved in the generation and migration of neural crest cells in embryonic stages which is driven by epithelial to mesenchymal transition (EMT). Presence of neuroendocrine cells in nests - neuroendocrine differentiation (NED) is a known histological marker for prostate Cancer. SNAI2 expression is down regulated in prostate cancer and silencing of the gene may turn on neuroendocrine differentiation, pluripotent genes and turn on specific metastasis suppressors [52]. SNAI2 knockdown initiating metastatic suppressor genes involves many pathways and further research is needed to derive a conclusion. Studies of SNAI2 gene regulation properties will help us in understanding the development of prostate cancer.

MRI1, Methylthioribose-1-Phosphate Isomerase 1 gene helps in catalyses of methionine, an important amino acid, in methionine salvage pathway. Development of certain cancers like prostate, glioma, bladder, breast, melanoma are dependent on methionine [53, 54]. To understand the dependency of methionine in prostate cancer a study has been conducted on patients who were not receiving any conventional treatment and were undergoing an intensive lifestyle program with a restricted methionine vegan diet. Analysis of serum samples revealed that there was a 70% inhibition of the growth androgen sensitive prostate adenocarcinoma (LNCaP) cells [55]. The data suggests that methionine restricted diet and lifestyle changes may help in slowing down the development of prostate cancer.

## Conclusion

Understanding gene activity in the prostate cancer laterality my help to guide the diagnosis and treatment of the disease. In this work, we have proposed a machine learning method that is capable of predicting with a high accuracy the tumor location in a cancer infected prostate. As a result, we have found genes as indicators that can differentiate the three locations of prostate cancer with high accuracy. The contributions of this study are two-fold. The proposed machine learning system can be used as a protocol for other types of cancer and other clinical problems in cancer studies. It also open the doors for potential biomarkers that can be further tested in wet-lab scenarios with the hope to move to clinical trials in order to replace the invasive biopsy or inaccurate image scanning.

The literature shows strong relations between prostate cancer metastasis and the computationally derived genes. Wet-lab experiments and RNA-seq profiling of those genes will better explore the relation between the findings and the prostate cancer laterality, which will potentially help the prognosis of the disease.

## Supplementary information

**Additional file 1** Bilateral-vs-Rest gene’s expressions.

**Additional file 2** Left-vs-Rest gene’s expressions.

**Additional file 3** Right-vs-Rest gene’s expressions.

## Data Availability

The datasets analyzed during the current study are available in the cBioPortal repository at http://www.cbioportal.org/study?id=prad_tcga.

## Abbreviations

Genes that can predict tumors in each location class of the prostate tumor. AUC: Area Under the Curve
DRE: Digital Rectal Exam
CRF: Conditional Random Field
ENN: Edited Nearest Neighbor
ER: Endoplasmic Reticulum
FBXO21: F-BOX Protein 21
HIF: Hypoxia-Inducible Fact
IG: Information Gain
MAGE: Melanoma-Associated Antige
MRI: Multiparametric Magnetic Resonance Imaging
mRMR: minimum Redundancy Maximum Relevance
MRP: Mitochondrial RNA Processing
NCL: Neighborhood Cleaning
NDUFA5: NADH:Ubiquinone Oxidoreductase Subunit A5
PP2A: Protein Phosphatase 2A
PRAD: Prostate Adenocarcinoma
PSA: Prostate Specific Antigen
RBF: Radial Basis Function
ROC: Receiver Operating Characteristic curve
SMOTE: Synthetic Minority Oversampling Technique
SRSF6: Serine and Arginine Rich Splicing Factor 6
SVM: Support Vector Machine
TCGA: The Cancer Genome Atlas
TPM: Transcripts Per kilobase Million
TRUS: Transrectal Ultrasound
